# Laparoscopic colonic resection for splenic flexure cancer: our experience

**DOI:** 10.1186/s12876-015-0301-7

**Published:** 2015-07-07

**Authors:** Andrea Pisani Ceretti, Nirvana Maroni, Matteo Sacchi, Stefano Bona, Maria Rachele Angiolini, Paolo Bianchi, Enrico Opocher, Marco Montorsi

**Affiliations:** 1 Department of General Surgery II, Ospedale San Paolo, University of Milan, Milan, Italy; 2 Department of General Surgery, Istituto Clinico Humanitas, IRCCS, University of Milan, Milan, Italy; 3 Department of General Surgery, Istituto Europeo di Oncologia, University of Milano, Milan, Italy; 4 Ospedale San Paolo, via Di Rudinì 8, 20142 Milano, Italy

**Keywords:** Laparoscopic resection, Colon cancer, Splenic flexure, Intracorporeal anastomosis

## Abstract

**Background:**

The treatment of colon cancer located in splenic flexure is not standardized. Laparoscopic approach is still considered a challenging procedure. This study reviews two Institutions experience in laparoscopic treatment of left colonic flexure cancer. Intraoperative, pathologic and postoperative data from patients undergoing laparoscopic splenic flexure resection were analyzed to assess oncological safety as well as early and medium-term outcomes.

**Methods:**

From October 2005 to May 2014 laparoscopic splenic flexure resection was performed in 23 patients.

**Results:**

Conversion rate was nihil. In 7 cases the anastomosis was performed intracorporeally. Specimen mean length was 21.2 cm, while the distance of distal and proximal resection margin from tumor site was 6.5 and 11.5 respectively. The mean number of harvested lymph nodes was 20.8. Mean operative time was 190 min and mean estimated blood loss was equal to 55 ml. As regard major postoperative complications, one case of postoperative acute pancreatitis and one case of postoperative bleeding from the anastomotic suture line were reported.

**Conclusions:**

Although our experience is limited and appropriate indications must be set by future randomized studies, we believe that laparoscopic resection with intracorporeal anastomosis appears feasible and safe for patients affected by splenic flexure cancer.

## Background

Laparoscopic surgery of colon cancer has been the subject of great interest since the first reports in 1991 [1, 2]. The most important prospective trials have revealed no differences between laparoscopic and open surgery in terms of lymph node harvest and resection margins clearance. They also clearly showed the short-term advantages of the laparoscopic approach, including less postoperative pain, improved respiratory function, early canalization and shorter hospital stay [3–6]. However, all these studies excluded patients with transverse colon and splenic flexure lesions, probably because of technical difficulties specific to this location, as identification of middle and left colic vessels and anastomosis construction. Ultimately, splenic flexure location has never been included in randomized controlled trials designed to assess the efficacy of laparoscopic surgery as a curative treatment for colon cancer. For this reason the treatment of cancer of the splenic flexure is not standardized, and the minimally invasive approach, especially if totally laparoscopic, is still considered very challenging. The main controversies include the appropriate extent of colon resection and lymph node dissection, the risk of inadvertent splenectomy and the type of anastomosis [7]. Aim of this study is to review our experience in laparoscopic treatment of splenic flexure tumors and to compare our data to the more recent literature.

## Methods

Splenic flexure cancer was defined as a tumor located in the distal third of the transverse colon, or in the left colonic angle, or in the proximal descending colon within 10 cm from the flexure [8]. From October 2005 to May 2014 minimally invasive approach was proposed to all patients with histological diagnosis of splenic flexure carcinoma, including patients with previous abdominal surgery or obesity. Exclusion criteria were totally obstructing tumors and locally advanced cancers (T4b). In patients with advanced stage at diagnosis or bulky disease, an open resection was the operation of choice; in case of adjacent organ involvement an en-bloc resection was performed. Diagnosis was made by colonoscopy and biopsy in all patients. Cancer staging was realized with thoracic and abdominal CT scan. Precise preoperative localization of the tumor was considered mandatory for laparoscopic resection planning. During traditional colonoscopy endoscopic tattooing with indian ink was performed in all patients. The CT scan was completed by virtual colonoscopy in 7 patients in order to improve cancer localization, to study the proximal colon in cases of non complete endoscopic exam and to evaluate the descending colon lenght.

Data regarding each patient entering the study were retrospectively collected and stored in a computerized database designed specifically to record the safety of laparoscopic colon surgery and follow the short- and medium-term outcomes. The clinical parameters we recorded included preoperative patient characteristics as demographics, body mass index (BMI), American Society of Anesthesiologists (ASA) score, previous surgical history. Intraoperative data included operative time, blood loss, conversion rate, length of skin incision, use of abdominal drains; operative time was calculated as the time between pneumoperitoneum induction and port-site closure; blood loss was measured by subtraction of the liquids instilled from those aspirated. Postoperative complications occurrence, length of hospital stay pathological report and short- and medium-term outcomes were retrospectively recorded. Pathologic examination confirmed type, grade and stage of the disease (according to AJCC/TNM), tumor diameter, number of harvested lymph nodes and length of the specimen. All patients were evaluated in outpatient setting 30 days after discharge. Those patients with cancer stage III were referred to the medical oncologist to be assessed for adjuvant chemiotherapy. All patients were followed up with a six month interval for five years and evaluated with physical examinaton and blood exams including serum hemoglobin, liver enzymes and tumor markers. A body CT scan was performed at least annually. Follow-up colonoscopy was recommended within one year of surgery.

This study received approval from our local Ethical Committee (Ethical committee of “Ospedale San Paolo”, via di Rudinì, 9 Milano – Italy).

### Surgical technique

**Fig. 1:**
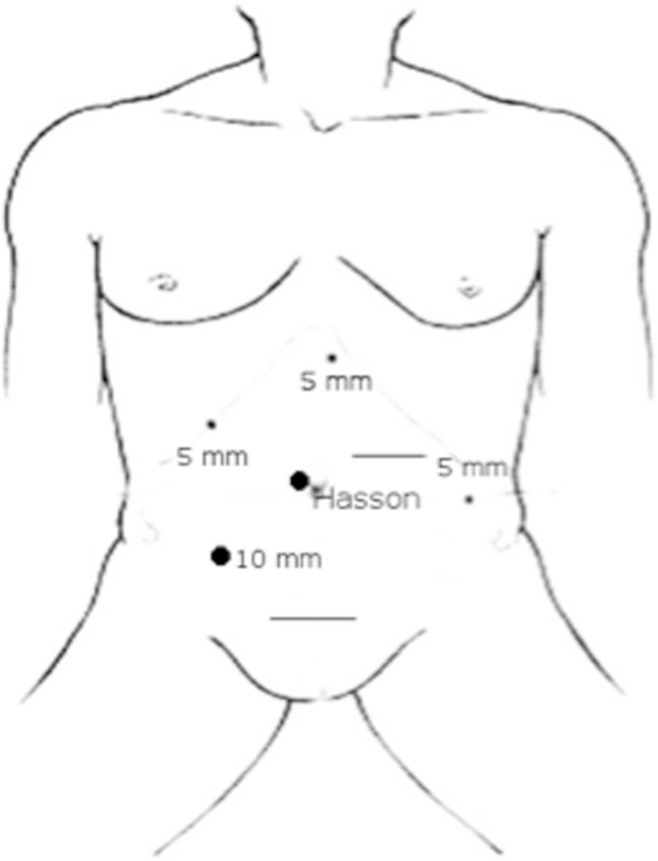
Trocar positions

**Fig. 2:**
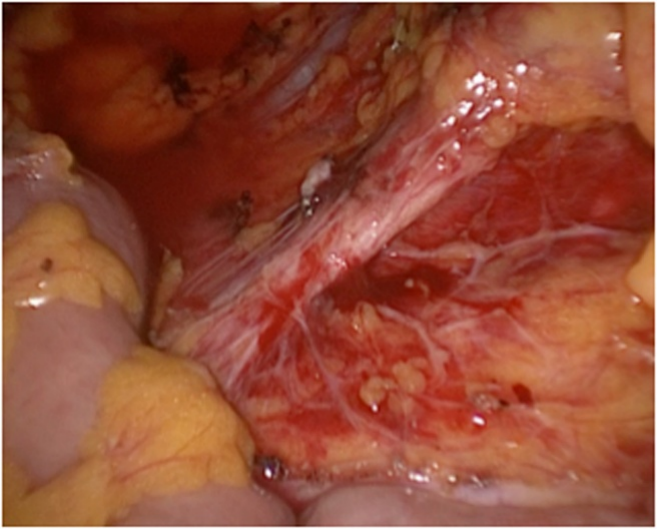
Division of left colic artery at his origin from inferior mesenteric artery

**Fig. 3:**
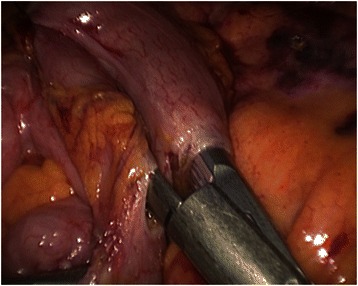
Intracorporeal stapling of side to side colo-colic anastomosis

**Fig. 4:**
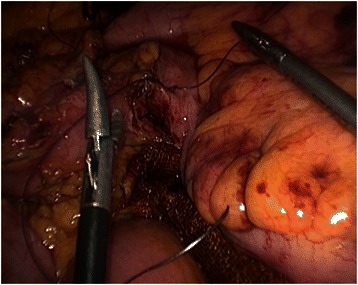
Closure of the enterotomy with intracorporeal suture and knotting

Informed consent was obtained from all patients. No patient received mechanical oral bowel preparation. All patients received perioperative antimicrobial (Cefuroxime and Metronidazolo) and antithrombotic prophylaxis. An urinary catheter was placed at the beginning of each procedure. All procedures were performed by three surgeons with proven experience in laparoscopic colorectal surgery. We adopted classical Lloyd-Davis position with both patient arms along the body. The operator and the first assistant were placed on the patient right side, with the second assistant between patient’s legs. The laparoscopic tower was on the left of the patient. The patient was kept in anti-Trendelenberg position and tilted 20 degrees rightward during the whole procedure in order to keep the operative field clean from small bowel loops. Four to five trocars were placed. The open technique was used to insert a 10–12 mm trocar on the umbilicus right side to introduce a 30 degrees scope. After pneumoperitoneum induction insufflation was maintained at 12 mm Hg. A 12 mm trocar was placed in the right lower quadrant for the operator right hand. A 5 mm trocar was inserted in the right hypocondrium for the operator left hand. A second 5 mm trocar was placed on the left side. A third 5 mm trocar could be added if necessary in the subxiphoid region. Trocars position is illustrated in Fig. 1. The primitive root of left mesocolon was incised from bottom to top, starting at the promontory and arriving at the duodenojejunal juncture. After inferior mesenteric artery identification, left colic artery was isolated and tied up at its origin (Fig. 2). The left Toldt fascia was dissected free from the prerenal fascia, from medial to lateral. Inferior mesenteric vein was identified close to the inferior pancreatic edge and closed off between clips. Transverse mesocolon was divided right to left along the inferior pancreatic edge, lowering the splenic flexure of the colon. The left paracolic gutter was incised bottom to top, joining the previous dissection of the left Toldt fascia. Division of splenocolic and gastrocolic ligaments from left to right completed splenic flexure mobilization releasing the distal third of transverse colon. The great omentum was divided using Harmonic scalpel and its left part was removed en bloc with the splenic flexure. The left branch of middle colic artery was ligated and divided as well as the ascending branch of the first sigmoid artery. Finally, descending colon was transected by linear stapler. Through a trasverse mini-laparotomy in left hypocondrium the colon was extracted and an extracorporeal double layer manual colo-colic anastomosis was performed. In the last two years, once we improved our laparoscopic technical skills and taking advantage of the experience we gained from right hemicolectomy, we began to performe intracorporeal anastomosis. In the latter case, the transverse colon was transected by linear stapler and an isoperistaltic side-to-side completely intracorporeal stapled anastomosis was maked (Fig. 3). The remaining enterotomies were closed in a double layer continue intracorporeal suture (Fig. 4). The specimen was routinely extracted through a suprapubic mini-laparotomy. In all cases we extracted the specimen using an abdominal wall protection device.

## Results

From October 2005 to May 2014 a minimally invasive approach was proposed to 23 patients affected by splenic flexure cancer. This group of patients corresponds to 3.9 % of global 586 laparoscopic resections for colo-rectal carcinoma we carried out in the same period, and the 76.6 % of all splenic flexure resections. As regard demographics, mean patients age was 70 ± 10.5 years (range 48–85); the male:female ratio was 9:14. Three patients were totally asymptomatic and received cancer diagnosis during our colorectal cancer screening regional program. Patients characteristics are summarized in Table 1. Conversion rate was nihil. We had no intraoperative complications. Intraoperative colonoscopy for tumor localization has never been necessary. In 7 patients the anastomosis was entirely intracorporeal. Mean operative time was 190 ± 49 min (range 150–295). In the group of patients undergoing extracorporeal anastomoses mean operative time was 182 ± 43 min, while the mean length of the mini-laparotomy was 8 ± 2 cm; on the opposite side, among patients undergoing the entire laparoscopic procedure mean operative time was 250 ± 50 min, while the mean length of abdominal incision was 5 ± 1 cm. The estimated blood loss was on average 55 ± 33 ml. Overall morbidity was 8.7 % (two patients), including a case of postoperative acute pancreatitis and a patient who experienced bleeding from the anastomosis stapler line in the first postoperative day. In the first case relaparotomy was necessary. In the second one the patient was treated by endoscopic hemostasis with clips placement. 30-days mortality rate was 0 %. Mean length of hospital stay was 8 ± 1 days (range 3–36). For the totally laparoscopic group the mean length of hospital stay was 8.0 ± 1.8 days (range 7–12) and for the patients undergoing extracorporeal anastomoses was 8.7 ± 7.5 days (range 3–36). The data of the intracorporeal and extracorporeal anastomosis are summerized in Table 2. The mean length of surgical specimen was 20.3 ± 4.3 cm (range 15–32), tumor diameter was 3.8 ± 2.3, the distance between proximal and distal margin from tumor site was 6.5 ± 2.1 and 11.5 ± 3.7 cm respectively. The mean number of harvested lymph nodes was 20.8 ± 5.3 (range 13–40). All intraoperative, postoperative and pathologic data are summarized in Table 3. No patient was lost during the observation period. The mean follow-up was 33 ± 17 months (range 5–96). With regard to oncologic outcome, one patient developed distant disease with liver metastases, while another one showed local recurrence in the anastomotic area. The primitive disease stage was advanced in both cases (pT3, N2a). In the first case the patient underwent liver resection after adjuvant chemioterapy. In the second case a further colic resection was performed; at the present time the patient is alive with no evidence of disease recurrence. Long-term mortality in term of global mortality and disease-specific was 0 %. Regarding long-term complications, a trocar site incisional hernia occurred in 1 case.

**Table 1:** Demographic data and stage distribution

Patients (n.)	23
Age (years)	70 ± 10.5
Sex (male/female)	9/14
BMI (Kg/m^2^)	24.4 ± 5.2
ASA [1–3]	10-10-3
Tumor site	
Distal transverse	6
Splenic flexure	13
Proximal descending	4
TNM stage (I, II, III)	9-8-6

**Table 2:** Comparison between totally laparoscopic operation and extracorporeal anastomosis

	Intracorporeal anastomosis	Extracorporeal anastomosis
Patients (n.)	7	16
Mean operative time (min)	250 ± 50	182 ± 43
Lenght of the minilaparotomy (cm)	5 ± 1	8 ± 2
Hospital stay (days)	8.0 ± 1.8	8.7 ± 7.5

**Table 3:** Intraoperative, postoperative and pathologic data

Tumor diameter (cm)	3.8 + 2.3
Distal margin (cm)	11.5 ± 3.7
Proximal margin (cm)	6.5 ± 2.1
Harvested nodes (n)	20.8 ± 5.3
Operative time (min)	190 ± 49
Blood loss (ml)	55 ± 33
Hospital stay (days)	8.5 ± 6.3
Conversions	0 %
Morbidity	8.7 %
30-days mortality	0 %
Follow-up (months)	33 ± 17

## Discussion

Splenic flexure carcinoma is a rare condition, as it represents approximately 3 to 8 % of all colon cancers. It is associated with high risk of obstruction and poor prognosis [9]. Surgical approach selection for splenic flexure carcinoma is still under debate. Similarly to other colon cancer sites, the resected area must encompass the mesocolon and include major vessels ligation at the origin; the rationale is to reduce local recurrence by complete removal of potentially involved lymph node stations. A more accurate definition of splenic flexure cancer may regard any lesion occurring between the distal third of the transverse colon and the first part of the descending colon [8]. The two major vessels which nourish colon splenic flexure are middle and left colic arteries. For these reasons the resection of a carcinoma located in one of these sites should always include lymphadenectomy up to the origin of superior and inferior mesenteric vessels, respectively. This could ultimately be the reason why splenic flexure cancers have never been included in randomized controlled trials, since their resection implies some technical difficulties, including laparoscopic identification of middle and left colic vessels and subsequent lymph node dissection. COST, COLOR, CLASICC and Barcellona trials [3–6] actually excluded patients with such lesions, and a future randomized clinical study specific for this subgroup appears really unlikely. For all these reasons laparoscopic treatment of splenic flexure carcinoma is still considered challenging, and clinical evidence of equivalence with other colon resections is still needed. Different procedures have been described in literature. Some Authors suggested to perform an extended right hemicolectomy performed with laparoscopic hand-assisted approach [10]. On the contrary, several Authors recommended a left partial colectomy with ligation at their origin of both left branch of the middle colic artery and left colic artery [11]. On the other hand, some investigators demonstrated that splenic flexure cancers are not associated with a worse prognosis compared to other colonic tumors and that the double lymphatic drainage does not confer a survival disadvantage, so that an extended resection appears unnecessary [12]. As described by some Authors, the oncological effectiveness of a segmental resection could be determined by the peculiar lymphatic spread of splenic flexure cancers: these studies showed that the majority of positive lymph nodes among patients with splenic flexure carcinoma are distributed along paracolic arcade and left colic artery. Nodes along middle colic artery and its left branch resulted involved in a negligible number of cases (0 and 4.2 %, respectively), thus not influencing the oncological outcome [13]. Therefore, a segmental resection can be effective for the treatment of splenic flexure cancer in its earlier phases. Moreover, laparoscopic segmental splenic flexure resection can be safely completed without identification and isolation of the middle colic vessels [11]. In fact, these Authors report that laparoscopic division of middle colic vessels is challenging as it requires advanced skills. In our series all patients underwent ligation at the origin of both middle colic artery left branch and left colic artery. Nevertheless, there were no significant differences in complication or conversion rates compared to patients who underwent laparoscopic resection of other colic segments [14]. Other main controversies making laparoscopic splenic flexure resection a a not yet standardized procedure concern the risk of inadvertent splenectomy and the type of intestinal anastomosis. As regard the first one theme, according to literature the risk of accidental splenectomy is higher in splenic flexure tumors compared to other colon cancer locations, thus leading to higher postoperative morbidity and mortality [7]. A mini-invasive approach proved to be especially suitable for splenic flexure mobilizing; due to its fixed position, some Authors suggested the use of Da Vinci system for this subgroup of colic cancer. Effectively, laparoscopic robot-assisted resection seems to be a promising approach for splenic flexure cancer treatment, since it allows finer manipulation which can decrease the risk of spleen injury [15]. Anastomosis is generally side-to-side performed. No data are available in literature comparing extracorporeal and intracorporeal anastomosis after laparoscopic splenic flexure resection and regarding immediately recognizable benefits for the patients and cost-effectiveness of the procedure. The majority of series suggests extacorporeal anastomosis; however, entirely intra-abdominal colon segments resection and anastomosis may become the procedure of choice [16]. Some of the potential advantages of intracorporeal anastomosis are the following: to anastomose away from the abdominal wall could reduce surgical-site infection rates; the reduced surgical manipulation of abdominal cavity may reduce adhesions and risk of adhesive small bowel obstruction; a smaller incision of abdominal wall for specimen extraction could lead to clinically relevant benefits; at last, laparoscopic visualization during the creation of the anastomosis could reduce unrecognized anastomotic twisting [17]. In our series an intracorporeal anastomosis was performed in the last 7 cases, after the improving of the surgical skill regarding intracorporeal sutures and knotting, using the same standardized technique of right hemicolectomy. In patients undergoing total laparoscopic treatment operative time was longer, but we didn’t record any case of anastomotic failure. A fast track protocol was performed in these patients, with good outcome in term of short terms complications and with shorter hospital stay. Preservation of inferior mesenteric and middle colic arteries could account for the good anastomotic healing, much more than improved experience with this technique [15]. Attemping to answer to all these controversies, we believe that when laparoscopic splenic flexure resection is performed after an adequate learning curve regarding other colon cancer locations, and if it is performed on appropriate patient groups with accurate preoperative diagnosis, this procedure should be considered a safe and useful treatment [18]. Indeed, in our series too, mortality was 0 % and 30-day morbidity resulted 8.7 %. The only major complication was a episode of postoperative acute pancreatitis involving pancreatic tail. This event simulated an anastomotic leakage, thus relaparotomy was necessary. Some Authors described postoperative acute pancreatitis occurring after surgical procedures involving transverse mesocolon root separation from pancreas [14]. Regarding oncological safety, this technique requires long-term follow-up observation to assess distant metastases and local recurrence rates; larger scale multicenter prospective studies on laparoscopic splenic flexure resection are therefore necessary. However, in our study a tumor-free resection margin was reported in all specimens and tumor distance from proximal and distal margins was always adequate. Lymphadenectomy was sufficient in all cases, too. During a mean follow-up of 33 ± 17 months one patient developed systemic recurrence with liver metastasis, while another one experienced a local recurrence in the anastomosic area, in accordance with a previous study describing an overall recurrence rate equal to 8.5 % [18]. Recurrent disease in these 2 cases was however related with the anatomopathological stage of the primitive disease (pT3, N1/2).

## Conclusion

Although our experience is limited and appropriate indications must be set by future studies, we believe that laparoscopic resection can be feasible and safe for patients with early-stage splenic flexure cancer. In a setting of surgeons experienced in laparoscopic colorectal surgery, the outcomes of laparoscopic splenic flexure cancers resection are similar to those of laparoscopic resections for cancer in other locations. The initial experience in totally laparoscopic approach with intracorporeal anastomosis appears promitting. This technique needs to be confirmed by multicentric prospective studies and in larger cohort of patients, especially when applied in fast track setting, where it appears to be particularly convenient.

## References

[1] Fowler DL, White SA (1991). Laparoscopy-assisted sigmoid resection. Surg Laparosc Endosc.

[2] Jacobs M, Verdeja JC, Goldstein HS (1991). Minimally invasive colon resection (laparoscopic colectomy). Surg Laparosc Endosc.

[3] Clinical outcomes of Surgical Therapy Study Group (2004). A comparison of laparoscopic assisted and open colectomy for colon cancer. N Engl J Med.

[4] Guillou PJ, Quirke P, Thorpe H, Walker J, Jayne DG, Smith AM, Heath RM, Brown JM, MRC CLASICC trial group (2005). Short-term endpoint of conventional versus laparoscopic assisted surgery in patients with colorectal cancer: multicentre, randomised controlled trial. Lancet.

[5] Lacy AM, Garcia-Valdecasas JC, Delgado S, Castells A, Taura P, Pique JM, Visa J (2002). Laparoscopy-assisted colectomy versus open colectomy for treatment of non-metastatic colon cancer: a randomised trial. Lancet.

[6] Veldkamp R, Kuhry E, Hop WC, Jeekel J, Kazemier G, Bonjer HJ, Haglind E, Pahlman L, Cuesta MA, Msika S, Morino M, Lacy AM (2005). Colon cancer Laparoscopic or Open Resection Study Group (COLOR). Laparoscopic surgery versus open surgery for colon cancer: short-term outcomes of a randomised trial. Lancet Oncol.

[7] McGory ML, Zingmond DS, Sekeris E, Ko CY (2007). The significante of inadvertent splenectomy during colorectal cancer resection. Arch Surg.

[8] Steffen C, Bokey EL, Chapuis PH (1987). Carcinoma of the splenic flexure. Dis Colon Rectum.

[9] Levien DH, Gibbons S, Begos D, Byrne DW (1991). Survival after resection of carcinoma of the splenic flexure. Dis Colon Rectun.

[10] Chew SS, Adams WJ (2007). Laparoscopic hand-assisted estended right hemicolectomy for cancer management. Surg Endosc.

[11] Kim CW, Shin US, Yu CS, Kim JC (2010). Clinicopathologic characteristics, surgical treatment and outcomes for splenic flexure colon cancer. Cancer Res Treat.

[12] Secco GB, Ravera G, Gasparo A, Percoco P, Zoli S (2007). Segmental resection, lymph nodes dissection, and survival in patients with left colon cancer. Hepatogastroenterology.

[13] Nakagoe T, Sawai T, Tsuji T, Jibiki M, Ohbantake M, Nanashima A, Yamaguchi H, Yasutake T, Kurosaki N, Ayabe H, Ishikawa H (2001). Surgical treatment and subsequent out come of patients with carcinoma of the splenic flexure. Surg Today.

[14] Schlachta CM, Mamazza J, Poulin EC (2007). Are transverse colon cancers suitable for laparoscopic resection?. Surg Endosc.

[15] Ceccarelli G, Biancafarina A, Patriti A, Spaziani A, Bartoli A, Bellochi R, Codacci Pisanelli M, Casciola L (2010). Laparoscopic resection with intracorporeal anastomosis for colon carcinoma located in the splenic flexure. Surg Endosc.

[16] Bergamaschi R, Arnaud JP (1997). Intracorporeal colorectal anastomosis following laparoscopic left colon resection. Surg Endosc.

[17] Casciola L, Ceccarelli G, Di Zitti L, Valeri R, Bellochi R, Bartoli A, Barchieri F, Spaziani A, D’Ajello M (2003). Laparoscopic right hemicolectomy with intracorporeal anastomosis: tecnica aspects and personal experience. Minerva Chir.

[18] Ham K-S, Choi G-S, Park J-S, Kim HJ, Park SY, Jun S-H (2010). Short-term outcomes of a laparoscopic left hemicolectomy for descending colon cancer: retrospective comparison with an open left hemicolectomy. J Korean Soc Coloproctol.

